# The Clinical Impact of Switching Basal Insulin to Insulin Degludec in Patients With Diabetes in Saudi Arabia: A Retrospective One-Group Pretest-Posttest Design Study

**DOI:** 10.7759/cureus.32091

**Published:** 2022-12-01

**Authors:** Abdulhamid Althagafi, Mohannad Alshibani, Samah O Alshehri, Afnan Barahim, Haneen Alghamdi, Dania Alaslani, Aisha Qari, Abdulaziz Almulhim, Ahmad Alamer

**Affiliations:** 1 Pharmacy Practice Department, Faculty of Pharmacy, King Abdulaziz University, Jeddah, SAU; 2 Pharmacy, King Abdulaziz University, Jeddah, SAU; 3 Department of Pharmacy Practice, College of Clinical Pharmacy, King Faisal University, Al-Ahsa, SAU; 4 Department of Clinical Pharmacy, College of Pharmacy, Prince Sattam Bin Abdulaziz University, Alkharj, SAU

**Keywords:** body weight., hypoglycemia, diabetes mellitus, degludec, basal insulin

## Abstract

Background: Diabetes is a health problem that has an enormous and intolerable public health burden on the individual, family, and community. Diabetes affects nearly one-fifth of adults in Saudi Arabia and is expected to double by 2030.

Aim of the study: The study aims to evaluate the impact of switching patients from conventional basal insulin analogues to insulin degludec during a 90-day follow-up period.

Methods: This was a retrospective observational pretest-posttest cohort study conducted at King Abdulaziz University Hospital between June 2019 and August 2020. Adult patients with diabetes who switched their basal insulin to insulin degludec were included and evaluated for its impact on insulin doses, hemoglobin A1c (HbA1c), hypoglycemic events, and/or body weight changes during a 90-day follow-up period.

Results: Out of 718 patients, 107 patients were included in the study, with 60.7% being females and their mean (± SD) age was 62.2 ± 14.6 years. There was a significant decrease in the mean baseline of HbA1c from 9.2% to 8.7% after 90 days of follow-up (P<0.001). A statistically significant reduction was noted in the total insulin requirements from a baseline of 71.70 (± 42.4) units to 46.5 (± 29) units, P=0.001, after switching to insulin degludec. However, there were no statistically significant differences in the body weight from the baseline mean (± SD) of 80.5 kg (± 19.4) to 79.9 kg (± 19.9), P=0.68, after switching to insulin degludec. Lastly, there were no statistically significant differences in the reported hypoglycemic episodes from a baseline of 48.7% vs 37.3% after 90 days of follow-up (P = 0.166).

Conclusion: Switching to the novel insulin degludec conferred better blood glucose control and dose reduction. There was no increase in the frequency of hypoglycemic episodes or body weight.

## Introduction

Diabetes mellitus (DM) is a major health problem with an enormous and intolerable public health burden. As a long-term metabolic disorder, diabetes exerts major impacts on the individual’s physical and mental health [1], as well as on the family caring for a diabetic patient [2] and the community [3]. The International Diabetes Federation reported that globally, the prevalence of diabetes was estimated to be 9.3% in 2019, with a projected increase of 25% in 2030 and 51% in 2045 [4]. Earlier estimates of the prevalence of DM have revealed that the Middle East includes 5 of the 10 countries with the world’s highest prevalence of DM, with Saudi Arabia having a prevalence of 18.7% in 2010 and, by 2030, a projected rate of 18.9% [5]. A large body of literature has emphasized the fundamental importance of improved glucose control in the reduction of long-term complications, including macro- and microvascular complications, renal insufficiency, and hypoglycemia, as well as a reduction in the mortality rate in patients with type 1 and type 2 DM [6,7]. However, in real practice, it was estimated that less than one-third of patients with type 2 DM achieved the recommended target of glycated hemoglobin (HbA1c) of less than 7% [8], which was due to the accompanying hypoglycemia [9].

First-generation basal insulins, including insulin glargine and insulin detemir, were found to significantly reduce within-subject insulin level variations as well as the frequency of hypoglycemic attacks in type 1 and type 2 DM due to the long half-life of the novel approach [10]. Nonetheless, first-generation basal insulins did not provide consistent and sustained 24-hour coverage. Moreover, within-subject variations, as well as the risk of hypoglycemia, were not fully satisfactory [11,12]. There was a discrepancy between the randomized controlled trials that support basal insulins and the real-practice evidence studies [11]. On the other hand, insulin degludec is a novel ultra-long-acting basal insulin analogue with slow and continuous absorption and a uniform, peakless action over approximately 25 hours and a long duration of action of more than 40 hours [13]. Insulin degludec was first introduced into the US market in 2015 [14] and, shortly after, was registered in Saudi Arabia under the trade name Tresiba®, which was available to be prescribed as FlexTouch 100 units/milliliter solution for injection [15]. The agent provides optimum glycemic control for type 1 and type 2 DM with low nocturnal and overall hypoglycemia rates in type 2 DM [16]. Accumulating evidence from literature has ensured the safety profile of insulin degludec. Insulin degludec was proven to allow glycemic control to reach a target of glycated hemoglobin of 7% with a low frequency of nocturnal hypoglycemia, even with substantial variation in dosing intervals, thus allowing dosing schedule flexibility and stability of glycemic control [17].

Switching from other types of basal insulin (detemir and glargine) to insulin degludec showed a high level of glycemic control and significant lowering of overall and nocturnal hypoglycemia in type 1 and type 2 DM in a one-year observational study [18]. Furthermore, switching to insulin degludec was found to be cost-effective for patients suffering from frequent hypoglycemic attacks and those who needed dose flexibility [19]. However, data from Saudi Arabia on switching patients from long-acting insulin are scarce. A retrospective study by Alsofiani et al. reported the incidence of diabetic ketoacidosis (DKA) in type 1 DM patients using insulin glargine (U300) compared to insulin degludec (U100) after six months [20]. Although the focus was to evaluate the incidence of DKA, for which there was a difference between the two groups, they reported no change in HgbA1c between the two therapies (9.9 vs 9.8, respectively; P>0.05), which suggests that there were no clinical benefits between the two agents in this population. However, the same study reported that insulin glargine (U300) users had higher insulin dose requirements when compared to insulin degludec (U100) users [20]. A Japanese study revealed that switching to insulin degludec resulted in better glycemic control in 16 weeks with lower insulin dosing requirements [21]. Similarly, real-world data reported a significant reduction in HbA1c, which was achieved after switching to insulin degludec, irrespective of the type of diabetes and the type of prior long-acting insulin, and with lower insulin requirements [22].

The current study reports clinical data from an understudied population at a single clinical practice in Saudi Arabia. We aimed to evaluate the clinical impact of switching patients with DM from long-acting insulins (glargine or detemir) to insulin degludec during a 90-day follow-up period.

## Materials and methods

Study design and setting

This was a retrospective one-group pretest-posttest design study conducted in an outpatient clinic of the tertiary hospital, which has a capacity of 1200 beds and is one of the largest medical centers in the western region of the county. Our study included patients seen in the outpatient setting only. Approval was granted for this study (HA-02-J-008) by the institutional review board of King Abdulaziz University Hospital on January 24, 2021. In our report, we followed the checklist from Strengthening the Reporting of Observational Studies in Epidemiology (STROBE) [23].

Patient selection

A list of adult patients (≥18 years old) with type 1 and type 2 DM seen between June 2019 and August 2020 was obtained using electronic health records and then by running a medication prescription report.

Inclusion criteria

The following criteria were used to select eligible patients for the study: (i) adult patients (18 years of age or older); (ii) documented diagnosis of type 1 or 2 DM; (iii) on insulin detemir or glargine and switched to insulin degludec; (iv) availability of records concerning HbA1C analysis and weight at the index date and at least 90 days after switching to insulin degludec.

Exclusion criteria

Patients were excluded from the study based on the following criteria: (i) pregnancy; (ii) pediatric patients <18 years; (iii) lack of follow-up data at 90 days; (iv) significant missing data.

Data collection

Data extraction was carried out on a password-protected Excel sheet (Microsoft Excel, Microsoft® Corp., Redmond, WA) by trained pharmacy interns and was reviewed for inconsistency, logical ranges, and missing variables.

On the index date (baseline), demographics, clinically pertinent data such as home medication, comorbid conditions, blood pressure, fasting blood glucose level (FBG), HbA1c, reported hypoglycemic events, and baseline insulin dosages were collected. The outcome data were collected at the end of the 90-day follow-up period.

Outcomes

Primary Outcome

For the first outcome, the measurements of the levels of HbA1c were taken on the index date and 90 days after switching to insulin degludec.

Secondary Outcomes

As the secondary outcome, the body weights were measured on the index date and 90 days after switching to insulin degludec. In addition, from the clinical notes, the number of reported hypoglycemic episodes and the total insulin were taken on the index date and 90 days after switching to insulin degludec.

Definitions

The date of switching from another basal insulin analogue to insulin degludec was identified for each patient as the index date or baseline date.

It is worth noting that insulin prescription is subject to the physician’s preferences based on their acquired experience and familiarity with the chosen basal insulin. There is no consensus or formal criteria that determine the choice of one basal insulin over the other.

Statistical analysis

Data were analyzed using Statistical Package for the Social Sciences (SPSS) software (IBM SPSS Statistics for Macintosh, Version 25.0, Armonk, NY: IBM Corp). The data were analyzed using descriptive and inferential statistics. Continuous variables were described as means ± standard deviation (± SD), and categorical variables were described as percentages. For the primary outcome (i.e., HgA1C changes after commencing insulin degludec), a paired t-test was used. For the secondary outcome (i.e., weight changes after commencing insulin degludec), analysis of variance (repeated-measure ANOVA) was used. Normality and homogeneity testing was conducted using diagnostic plots (the normal Q-Q plot) and Leven’s test. P-values of less than 0.05 were considered statistically significant. For missing data, these were determined to be missing at random (MAR). Multiple imputations (5 imputed datasets with 10 iterations) [24] used the Markov chain Monte Carlo (MCMC) method with linear regression as the model type for scale variables. Convergence plots were generated to check for variations between the iterations.

## Results

**Figure 1:**
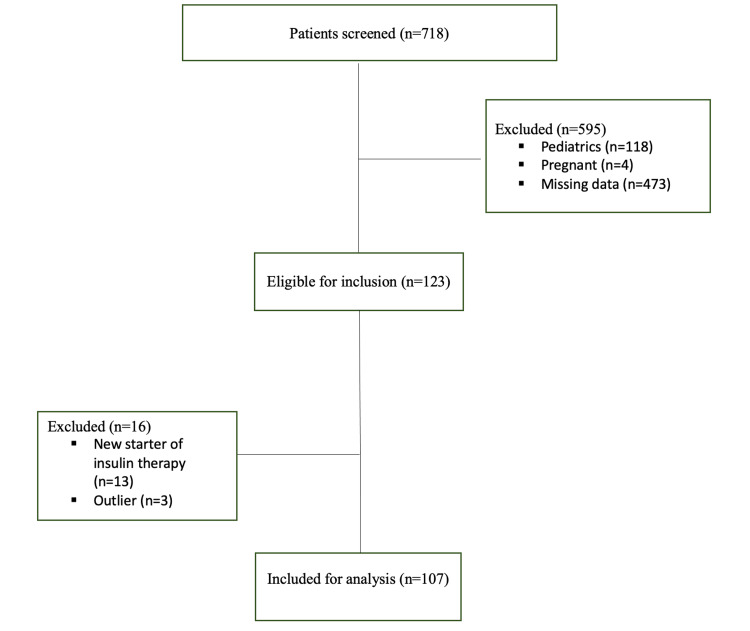
Patient selection flowchart

A total of 718 patients were screened, and, of these, 595 were excluded via the exclusion criteria, leaving 123 eligible patients. Only 107 patients were enrolled for data analysis after 16 patients were subsequently excluded (Figure 1). The majority of patients were female (60.7%) with a mean (± SD) age of 62.2 (± 14.6) years, and most of the patients had type 2 DM (72%). Before switching to insulin degludec, the mean (± SD) HbA1c was 9.2% (± 1.7), the mean body weight was 80.8 kg (± 19.4), and the mean (± SD) FBG was 169.6 mg/dl (± 66.5). The mean (± SD) total daily insulin dose was 71.70 units (± 42.4). In addition, the baseline mean (± SD) systolic blood pressure was 137 mmHg (± 19.5), and diastolic pressure was 72.2 mmHg (± 12.7). Reported hypoglycemic episodes were 16.8% at baseline. For comorbidities, 74% of the patients were found to have hypertension, 76% had dyslipidemia, and 35.5% had retinopathy. Statins were the most prescribed medication (82%), followed by metformin (52.3%) (Table 1).

**Table 1:** Clinical outcome data before and after switching to degludec

HbA1c, mean (95%CI)			
Before insulin degludec (n=107)	HbA1c after insulin degludec (n=107)	Mean change	P-value
9.1 (8.9–9.2)	8.5 (8.3–8.6)	0.56 (0.44–0.68)	<0.001
Insulin units, mean (95% CI)			
72.1 (68.7–75.4)	46.9 (44.7–49.1)	25.7 (23.2–28.2)	0.001

Switching to insulin degludec was associated with a significant decrease in the mean of HbA1c with a mean change of 0.56 (95% CI 0.44-0.68, P<0.001) at the end of the 90-day follow-up period. Switching to insulin degludec was not associated with a significant decrease in body weight, with weight at 90 days having a mean (SD) of 80.6 (18.4, P=0.68). There was a significant decrease in total insulin use with a mean change of 25.7 (95% CI 23.2-28.2, P=0.001) after switching to insulin degludec (Table 2). Hypoglycemic episodes were reported in 50 patients (48.7%) at baseline.

**Table 2:** Baseline characteristics of all patients on the index day T1DM: type 1 diabetes mellitus, T2DM: type 2 diabetes mellitus, HbA1c: glycated hemoglobin A1c, ARBs: angiotensin receptor blockers, BBs: beta blockers, DPP4Is: dipeptidyl peptidase 4 inhibitors, ACEIs: angiotensin-converting enzyme inhibitors, CCBs: calcium channel blockers, SGLT-2Ia: sodium-glucose cotransporter-2 inhibitor, GLP-1: glucose-like peptide-1.

Baseline characteristic	Value
Age, mean (±SD)	53.7 (14.6)
Female, n (%)	65 (60.7)
Type of DM, n (%)	
T1DM	19 (17.8)
T2DM	77 (72)
Weight, mean (±SD)	80.8 (19.4)
HgbA1c, mean (±SD)	9 (1.7)
Fasting plasma glucose, mean (±SD)	169.6 (66.5)
Systolic blood pressure, mean (±SD)	138 (19.5)
Diastolic blood pressure, mean (±SD)	72.2 (12.7)
Total daily insulin dose, mean (±SD)	74.5 (43.6)
Reported hypoglycemia, n (%)	18 (16.8)
Comorbidities, n (%)	
Hypertension	80 (74)
Dyslipidemia	76 (71)
Retinopathy	38 (35.5)
Nephropathy	31 (29)
History of acute coronary syndrome	24 (22.4)
Heart failure	9 (8.4)
Stroke	4 (3.7)
Peripheral artery disease	4 (3.7)
Medications, n (%)	31 (29)
Statins	82 (76.6)
Metformin	56 (52.3)
ACEIs	31 (29)
ARBs	44 (41)
BBs	36 (33)
Diuretics	30 (28)
Sulfonylureas	20 (18.7)
SGLT-2Is	19 (17.8)
GLP-1 agonists	8 (7.5)

For type 1 DM, there was a statistically significant reduction in the total insulin requirements, with a mean change of 25.5 (95% CI 20.3-30.7) and P ≤ 0.001 at follow-up after switching to insulin degludec. Similarly, type 2 DM showed a significant decrease in the mean total insulin requirements among patients, with a mean change of 25.5 (95% CI 22.5-28.5; P ≤ 0.001) (Table 3).

**Table 3:** Difference between total insulin requirements before and after degludec for patients with T1DM and T2DM

Type 1 DM (n=19)	Mean (±SD)	P-value
Insulin before degludec	77 (42.1)	<0.001
Insulin after degludec	51 (42.7)	
Type 2 DM (n=77)	Mean (±SD)	P-value
Insulin before degludec	70.6 (42)	<0.001
Insulin after degludec	45.8 (24.4)	

## Discussion

The study demonstrated marked glycemic benefits after switching the basal insulin of uncontrolled patients with diabetes (mean HbA1c of 9%) to insulin dugledac after a 90-day follow-up period. The improved glycemic control was accompanied by a reduction in the total daily dose requirements in these patients (mean change of 25.2 units). In agreement with our findings, a study by Fadini et al. from a large multicenter real-world setting in Europe in which patients’ basal insulin was switched to insulin deglude found a significant HbA1c reduction of 0.32% in type 2 diabetes as well as a reduction in insulin requirements at the 12-month follow-up period [25]. Similar findings were obtained in a Canadian population in an observational retrospective study where they included 626 patients who were switched to insulin degludec with a mean follow-up of six months and which resulted in a reduction of HgbA1c by 0.30 in both types of diabetes [26]. However, unlike our results, the study by Harris et al. [26] found a statistically significant reduction of insulin requirements only in type 1 DM and not type 2 DM.

The mean weight change in our study was not significant. This is perhaps due to the short follow-up period. However, in the RESTORE-1 retrospective study from Italy, in which patients were switched to either insulin glargine (U300) or dugladec insulin, no weight change was observed at the three- or six-month follow-up periods [26]. Interestingly, the same authors reported a slightly lower incidence of hypoglycemia with glargine U300 compared to dugladec insulin, though without statistical significance. Nonetheless, both Fadini et al. and Harris et al. found a significant reduction after switching to insulin dugladec [25,26].

Our study has a number of advantages. It was conducted in a large outpatient setting in Saudi Arabia, and, due to the lack of reports in the region, our study is the first to assess switching patients to insulin dugladec in Saudi Arabia. From a Saudi healthcare perspective, this creates the opportunity to conduct cost-effectiveness studies on switching uncontrolled diabetes patients to this agent. For instance, in a Spanish study, insulin dugladec was found to be the dominant strategy for treating type 2 diabetes patients when compared to insulin glargine. The cost benefits were mainly driven by the lower hypoglycemic events associated with the agent. We are unaware of economic studies conducted in our region from the Saudi healthcare perspective [27,28]; however, in a study abstract by Jamjoom, which was published in Value in Health, the authors compared the price of insulin dugledec in three countries in 2020 (prices were indexed to Canada = 100). For the 100U insulin dugledac pens, it was 63.8 in Saudi Arabia, 100 in Canada, and 385.1 in the US. These lower costs of insulin may be due to policies from the Saudi Food and Drug Administration that require a Certificate of Pharmaceutical Product (CPP) for review and completion of pricing negotiations before approval [29] and for marketing the drug in the country.

However, there were several limitations to our study. This was an observational one-group (lack of control group) retrospective study in a single region of Saudi. The study was heavily dependent on clinical notes. To avoid coding errors, we avoided administrative data, as it is sometimes prone to coding errors. The data were reviewed manually in order to be extracted and coded for analysis. Nonetheless, this cannot entirely rule out the potential of data collection bias [30]. Due to the limited number of patients identified in a one-year period who were prescribed insulin dugledec, we were forced to use all the data we had (convenience sampling). This non-probability sampling can lead to over-representation or underrepresentation of the population (in our case >60% were female patients). This can limit the generalizability of the study. Lastly, the use of patients’ records can mean important outcomes are not reported (namely hypoglycemia events), as we were dependent on the physicians’ clinical notes.

## Conclusions

In conclusion, in patients with type 1 and type 2 DM, switching to insulin degludec offered better glycemic control, which was manifested by a reduction in HbA1c levels, a reduction in insulin dose, and no increase in the frequency of reported hypoglycemia in this Saudi Arabian population. However, since this is a single-centered study conducted retrospectively, these findings should be supported by randomized control trials.
